# Action video games normalise the phonemic awareness in pre-readers at risk for developmental dyslexia

**DOI:** 10.1038/s41539-024-00230-0

**Published:** 2024-03-21

**Authors:** Sara Bertoni, Chiara Andreola, Sara Mascheretti, Sandro Franceschini, Milena Ruffino, Vittoria Trezzi, Massimo Molteni, Maria Enrica Sali, Antonio Salandi, Ombretta Gaggi, Claudio Palazzi, Simone Gori, Andrea Facoetti

**Affiliations:** 1 Università di Bergamo, Department of Human and Social Sciences, Bergamo, Italy; 2 Università di Padova, Developmental Cognitive Neuroscience Lab, Department of General Psychology, Padova, Italy; 3 Université Paris Cité, Laboratoire de Psychologie de Développement et de l’Éducation de l’Enfant (LaPsyDÉ), UMR CNRS 8240 Paris, France; 4 Università di Pavia, Department of Brain and Behavioral Sciences, Pavia, Italy; 5 Scientific Institute, IRCCS E. Medea, Bosisio Parini, Child Psychopathology Unit, Lecco, Italy; 6 Sigmund Freud University, Milano, Italy; 7 ASST Valle Olona, Neuropsychiatric Unit, Saronno, Varese Italy; 8 Università di Padova, Department of Math, Padova, Italy

**Keywords:** Dyslexia, Human behaviour, Attention, Sensory processing

## Abstract

Action video-games (AVGs) could improve reading efficiency, enhancing not only visual attention but also phonological processing. Here we tested the AVG effects upon three consolidated language-based predictors of reading development in a sample of 79 pre-readers at-risk and 41 non-at-risk for developmental dyslexia. At-risk children were impaired in either phonemic awareness (i.e., phoneme discrimination task), phonological working memory (i.e., pseudoword repetition task) or rapid automatized naming (i.e., RAN of colours task). At-risk children were assigned to different groups by using an unequal allocation randomization: (1) AVG (*n* = 43), (2) Serious Non-Action Video Game (*n* = 11), (3) treatment-as-usual (i.e., speech therapy, *n* = 11), and (4) waiting list (*n* = 14). Pre- and post-training comparisons show that only phonemic awareness has a significantly higher improvement in the AVG group compared to the waiting list, the non-AVG, and the treatment-as-usual groups, as well as the combined active groups (*n* = 22). This cross-modal plastic change: (i) leads to a recovery in phonemic awareness when compared to the not-at-risk pre-readers; (ii) is present in more than 80% of AVG at-risk pre-readers, and; (iii) is maintained at a 6-months follow-up. The present findings indicate that this specific multisensory attentional training positively affects how phonemic awareness develops in pre-readers at risk for developmental dyslexia, paving the way for innovative prevention programs.

## Introduction

Playing is essential in child development and it is present in humans from early childhood. Playing could represent the optimal enriched environment for cognitive development and learning^1^ by activating a specific combination of large-scale neural networks, including mesolimbic emotional and reward pathways^2^. Accordingly, play-driven neural, physiological and biochemical activation could boost attention, emotion regulation, visual object recognition and language development^3^. Nowadays, video-gaming is one of the most popular forms of playing in children, and a recent review tries to identify how common features between video-gaming and traditional game affect development and learning^4^. The impact of emotional and reward signals induced by video-gaming^5^, in combination with specific features of the gamified training, could induce far-transfer cognitive enhancements in (a)typical readers. For this reason not all video-games have the same impact on behaviour and the developing brain^6^.

Among the different genres of video-games, action video-games (AVGs) are an excellent tool for the investigation of human learning and neuroplasticity^7^. AVGs are characterised by high-speed events and fast-moving targets, high perceptual, cognitive and motor loads, emphasis on the peripheral visual field, and spatial and temporal unpredictability^8^. AVGs provide strong emotional and reward signals stimulating the motivated behaviour and are able to activate the attentional control, placing the brain in a more plastic state^9^. The attentional control combines the ventral stimulus-driven and the dorsal goal-directed fronto-parietal networks^10^, and allows to select both low-level (e.g., discrimination of acoustic frequencies processing) and high-level (e.g., rhyme and phonemic awareness) relevant information which is worthy to consolidate and automatize to increase the task’s performance^11^. Moreover, by stimulus-driven and goal-directed shifting of the information processing resources, the attentional control includes executive function mechanisms, such as inhibition, switching, and working memory^9,12^. Together with the reward systems, they lead to a more efficient processing and learning because they can attenuate the processing of goal-irrelevant information^9^.

The effects of AVGs upon neuroplasticity in spatial, temporal and object-based shifting of visual attention, have been consistently demonstrated^9,13–16^. A recent study involving 151 typical readers provided further evidence about the efficacy of AVGs^17^. In particular, after a video-game mixing action mechanics and executive functions training, the authors showed a significant improvement in visuo-spatial attentional shifting, planning skills and reading, lasting at the 6-months follow-up. Importantly, this video-game also improved language school grades^17^. Chaarani et al.^18^ showed that video-gamers exhibited faster inhibition and working memory compared to non-video-gamer children, and demonstrated differences in brain activations in key regions of the cortex responsible for visual, attention, and working memory processing. More stringently, the time spent at video-gaming longitudinally enhances children’s intelligence and reading skills development while controlling for the confounding effects of genetic differences in cognition and socioeconomic status^19^.

Interestingly, Green et al.^20^ showed that AVGs also enhance the efficiency of the auditory noise-exclusion mechanism as they lead to an increase in the rate at which sensory information is accumulated over time (“accumulation of evidence” or processing speed^21,22^). A recent cross-sectional study showed that AVGs accelerate attentional shifting during a stimulus-driven auditory-cueing task which is in turn linked to better phonological working memory and reading skills in adult AVG players^23,24^. The sum of these data support video-gaming and especially AVGs as an effective remediation tool in neurodevelopmental disorders^25–27^ characterised by visual and auditory attention deficits such as developmental dyslexia (DD^28–37^).

DD is a hereditary and severely invalidating learning disability that affects literacy acquisition despite typical intelligence and adequate education^38^. Reading is instrumental to civilization and to daily life and learning, so that DD is often associated with undesirable outcomes such as lower educational attainment and loss of self-confidence^39^. Although deficits in phonological processing have been consistently reported as associated with and predictive of DD^38–43^, it has been also argued that a basic visual or/and auditory disorder of attentional shifting with its noise-exclusion mechanism^9^, may be etiologically relevant for DD^44–50^. In particular, Hari and Renvall^45^ suggested that sluggish attentional shifting could account for the impaired processing of rapid stimuli, such as phonemic and syllabic perception, in DD and language-based learning disability^44,46,51,52^. Within this attentional-framework, the prolongation of the attentional shifting could be crucial in explaining many (if not all) perceptual and phonological difficulties with syllables, rhymes and phonemes found in children with DD and language-based learning disability. A sluggish attentional shifting would prolong sensory input chunks, thereby increasing the neural noise and degrading cortical representation of speech components essential for reading acquisition^12,30,45,46,50,52^. Auditory attentional shifting influences the learning trajectories of symbol-speech sound correspondence, modulating the neural sound tracking in children with and without DD^53,54^. A recent meta-analytic review demonstrated that children with DD showed deficits across processing speed, inhibition, switching, working memory and visuospatial skills, and that, after controlling for phonological processing and language comprehension, attentional control uniquely contributed to reading skills^55^. Interestingly, impaired attentional control in DD was associated with greater deficits in reading fluency and greater reductions of activations in response to print in the typical left-hemisphere phonological and orthographic reading networks^56^, supporting the relationship between the neurobiology of attentional control and reading^57^. Several longitudinal studies have shown that an auditory sluggish attentional shifting in pre-readers at-risk for DD, significantly predicted future reading impairments in primary school^58–60^. Finally, gamified attentional control (e.g., inhibition and shifting) training programs were shown to improve both reading performance and reading comprehension in typical readers^17,61–64^. As deficits in phonological processing have been proposed as the most accepted etiological mechanisms underlying DD^39^, remediation treatment-as-usual aims to train phonemic awareness and letter-to-sound knowledge through direct instruction in the context of high-quality phonologically based reading instruction^40,65–67^. However, although notes of caution were reported for the need of larger sample size and follow-up assessments, a recent review concluded that visual attentional interventions are effective for treating children with DD, improving reading fluency equal to or greater than other more traditional phonological programs^68^. An emerging field of digital health demonstrated that 12 h of AVGs were able to improve both visuo-spatial attentional shifting and reading fluency in a small group of Italian children with DD, providing a new, fast and fun remediation of DD^6^. These results showed that a visual attentional shifting improvement can directly translate into better reading abilities. This pioneering study was replicated in English-speaking children with DD who showed an improvement also in auditory-phonological working memory^69^. Other studies showed that AVGs led to an improvement in phonological working memory^35,70^ as well as in the processing speed of auditory cues alerting the display of the visual targets^6^ and of attentional shifting between auditory and visual modality^69^ in children with DD. Interestingly, the improvement in these neurocognitive skills significantly predicted reading fluency^6,69^, suggesting that an increased attentional shifting may lead to gains not only in letter-string discrimination, but also auditory, cross-modal and phonological processing. In a recent randomised controlled trial in English-speaking children with DD, a brief AVG training led to improvements in text reading rate and accuracy, reading comprehension as well as rapid automatized naming (RAN^71,72^). Finally, recent meta-analyses showed that AVGs training positively affects visual attention (g = 0.72) as well as reading speed (g = 0.44) and phonological processing (g = 0.45)^26,27^.

Taken together these findings pave the way for a new hypothesis: AVGs could boost auditory attentional shifting, phonemic awareness, phonological working memory, RAN and reading skills, because they could be all underlined by “accumulation of evidence”^20,23^. Accordingly, the sluggish attentional shifting^45^ could be equivalent to a slow “accumulation of multisensory evidence”^20^. The attention networks involved in the sluggish attentional shifting and “accumulation of sensory evidence” are often multimodal, such as the “When” pathway in the right inferior parietal lobe^73^, the right ventral fronto-parietal network of stimulus-driven control of attention^10^, the alerting system of right thalamic and fronto-parietal network^74^, and the right anterior insular cortex of salience network^75^. Interestingly, a recent functional neuroimage meta-analysis conducted on 96 studies in children with DD have shown an overactivation for reading in the right insula^76^. A mild developmental dysfunction of these right multimodal neural networks could be also linked to the “left-mini neglect” found in adults and children with DD^28^^,^^77–79^. Moreover, deficits in the multimodal neural networks have been shown to be associated with two DD-candidate genes^80–82^ (i.e., *DCDC2* and *ROBO1*), suggesting that the sluggish accumulation of sensory evidence in individuals with DD^83,84^ could be linked to genes involved in neuronal migration, neurite outgrowth and cortical morphogenesis^85^.

**Fig. 1:**
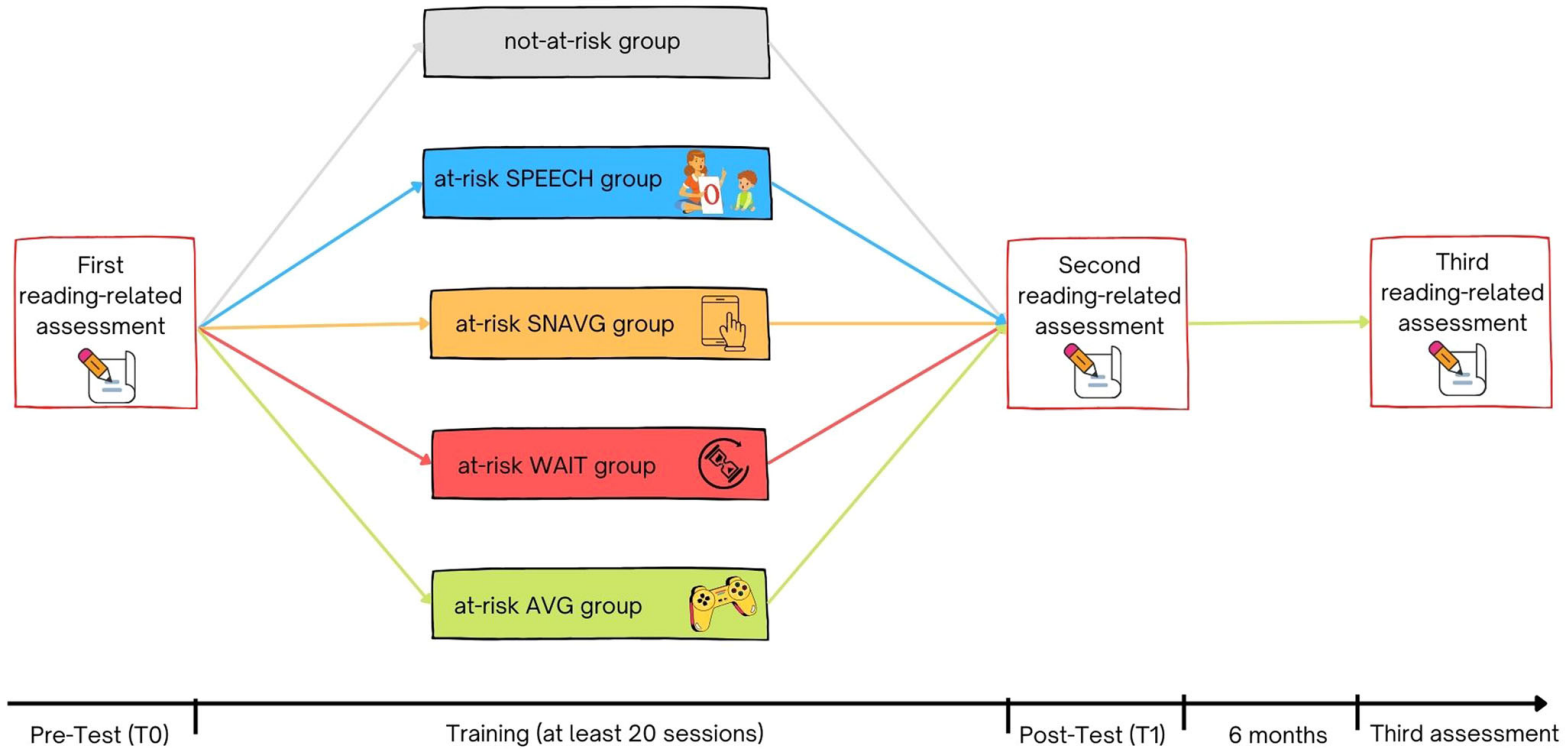
Representation of the timeline of the prevention study. Not-at-risk group included pre-readers with average scores in all the reading-related tasks (i.e., phonemic awareness, phonological working memory and rapid naming). At-risk groups included pre-readers who had at least −1.00 SD below the mean in at least one of the language-based tasks (i.e., phonemic awareness, phonological working memory, or rapid naming). WAIT waiting list, SNAVG Serious Non-Action Video Game, SPEECH Speech therapy (treatment-as-usual), AVG Action Video game.

According to the notion that AVGs improve the “accumulation of evidence”^20^, and by hypothesising that the phonological deficits can be explained by a sluggish “accumulation of evidence”^28,29,41,45,46,83,84,86^ in early ages, we tested whether deficits in the consolidated language-based predictors of reading development can be overcome with AVG training in pre-readers at-risk for DD. That is, the accelerating of auditory attentional shifting AVG-induced could enhance reading development predictors in pre-readers at-risk for DD and, potentially, reduce the subsequent development of DD. In this prevention study, we tested the efficacy of a commercial AVG upon three consolidated reading development predictors, i.e. phonemic awareness, phonological working memory and rapid naming^40,87,88^. One hundred and twenty pre-readers have been included in this study and grouped according to the scores in three reading-related tasks, i.e., phonemic discrimination (PD), pseudo-word repetition (PWR) and RAN of colours. This led to 79 pre-readers at-risk for DD (i.e., at least −1.00 SD below the mean in at least one of the above-mentioned reading-related tasks) and 41 not-at-risk pre-readers (i.e., average scores in all the above-mentioned reading-related tasks). At-risk children were assigned to different groups by implementing an unequal allocation randomization^89,90^: (1) AVG (*n* = 43); (2) Serious Non-Action Video Game (SNAVG; *n* = 11); (3) treatment-as-usual (i.e., speech therapy - SPEECH; *n* = 11); and (4) waiting list (WAIT; *n* = 14). We predicted the AVG group would show larger improvement in phonological skills than the WAIT, SNAVG and SPEECH groups. By affecting reward and attentional control mechanisms, the AVGs would accelerate the accumulation of evidence in at-risk pre-readers. In particular, we expected more robust group differences in PD compared to PWR and RAN tasks. Phonemic awareness can be considered more sensitive to the sensory accumulation of evidence^46^ compared to working memory and RAN, which involve complex cognitive, memory, cross-modal association and motor processes that are still developing in pre-readers. Accordingly, we tested whether AVGs could lead to (i) a catch-up in phonemic awareness, (ii) long-lasting effects, (ii) in most of the children who make this at-risk group up (see Fig. 1).

## Results

At-risk and not-at-risk children significantly differed for a socio-demographic variable, i.e. sex (Pearson’s chi-square = 6.325, df = 1, *p* = 0.012) showing a male:female ratio of 1.55 and 0.58, respectively. As expected, the two groups statistically differed for reading-related variables at pre-training (T0) also after controlling for sex (MANCOVA: F_(3,115)_ = 51.108, *p* < 0.001, η_p_^2^ = 0.571; ANCOVA upon PD: F_(1,117)_ = 44.510, *p* < 0.001, η_p_^2^ = 0.276; ANCOVA upon PWR: F_(1,117)_ = 73.458, *p* < 0.001, η_p_^2^ = 0.386; ANCOVA upon RAN: F_(1,117)_ = 21.766, *p* < 0.001, η_p_^2^ = 0.157).

### Effects of AVGs upon the reading-related deficits

Table 1 shows the descriptive statistics of the reading-related variables at T0 and T1 in the different training groups. The training groups did not differ either by sociodemographic variables (i.e., sex: Pearson’s chi-square=4.890, df = 3, *p* = 0.180; age: F_(3,75)_ = 0.411, *p* = 0.746, η_p_^2^ = 0.016; Block design subtest: F_(3,75)_ = 1.072, *p* = 0.366, η_p_^2^ = 0.041) nor by reading-related variables at T0 (i.e., PD, PWR and RAN; MANOVA F_(9,219)_ = 1.769, *p* = 0.075, η_p_^2^ = 0.068), nor by hours of treatment (F_(2,62)_ = 1.878, *p* = 0.161, η_p_^2^ = 0.057).

**Table 1:** Descriptive statistics of the phonological variables and hours of training in the different training groups before (T0) and after (T1) the training

			WAIT (*n* = 14)	SNAVG (*n* = 11)	SPEECH (*n* = 11)	AVG (*n* = 43)
T0	PD	Mean	−3.294	−0.919	−1.625	−2.102
		SD	2.343	1.542	1.271	1.874
		Min	−6.392	−3.155	−3.803	−6.392
		Max	0.728	0.728	0.081	0.728
	PWR	Mean	−1.363	−2.046	−2.426	−2.098
		SD	1.131	0.528	0.789	1.347
		Min	−2.866	−3.076	−3.916	−4.546
		Max	1.126	−1.185	−1.605	2.597
	RAN	Mean	−1.442	−2.422	−2.208	−2.280
		SD	3.158	3.792	1.348	2.513
		Min	−11.143	−11.746	−4.337	−11.716
		Max	1.237	1.810	0.059	1.040
T1	PD	Mean	−2.462	−0.919	−1.037	−0.431
		SD	2.454	1.306	1.265	1.344
		Min	−6.392	−3.803	−3.803	−5.097
		Max	0.728	0.728	0.728	0.728
	PWR	Mean	−0.660	−0.402	−1.471	−1.068
		SD	1.443	1.462	1.089	1.403
		Min	−3.076	−2.655	−3.076	−3.916
		Max	2.387	1.756	0.496	2.597
	RAN	Mean	−0.586	−1.074	−1.080	−0.906
		SD	1.465	1.525	1.038	1.380
		Min	−3.987	−4.228	−3.316	−4.500
		Max	1.659	0.663	0.059	1.028
Hours of training		Mean	/	19.909	22.136	19.512
		SD	/	0.302	6.565	3.673
		Min	/	19.000	16.000	15.000
		Max	/	20.000	31.000	24.000

*PD* Phonemic discrimination, *PWR* Pseudo-word repetition, *RAN* Serial rapid automatized naming.

**Fig. 2:**
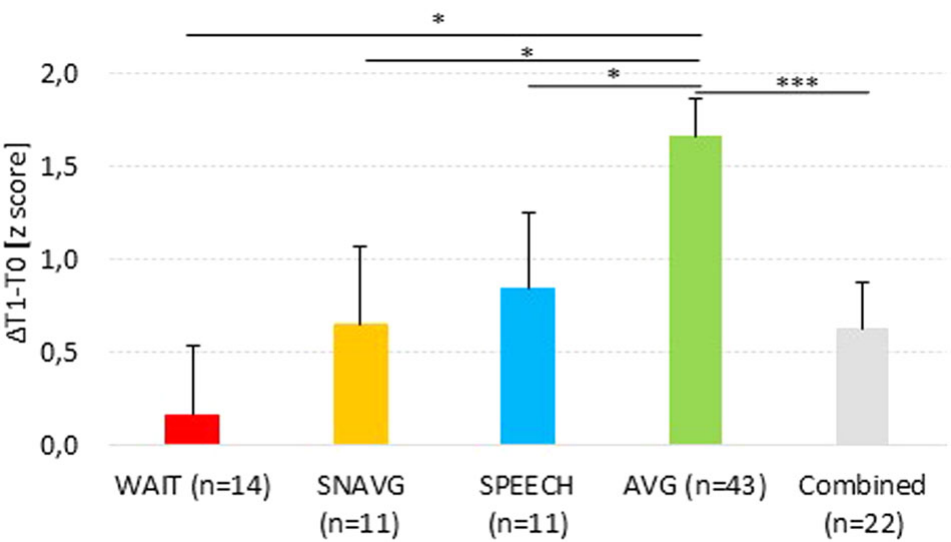
Effects of AVGs upon phonemic awareness. Mean of the individual gain between pre- and post-training (ΔT1-T0). WAIT Waiting list, SNAVG Serious Non-Action Video Game, SPEECH Speech therapy (treatment-as-usual), AVG Action Video game; Combined SNAVG and SPEECH. Error bars represent standard error of the mean. *p < 0.05 and ***p < 0.01.

A statistically significant different individual gain (ΔT1-T0) between groups was found in PD (F_(3,74)_ = 5.175, *p* = 0.003, η_p_^2^ = 0.173). The AVG training led to higher individual gains (mean=1.660, 95% CI = 1.248/2.072) compared to the WAIT (mean = 0.160, 95% CI = −0.590/0.910), the SNAVG (mean = 0.645, 95% CI = −0.192/1.482), and the SPEECH (mean = 0.842, 95% CI = −0.024/1.659) groups (Fig. 2). Post-hoc analysis for 1,000 bootstrap resamples showed that individual gain after the AVG training was significantly higher compared to the WAIT (mean difference = 1.500, 95% CI = 0.391/2.785, *p* = 0.014), the SNAVG (mean difference = 1.015, 95% CI = 0.283/1.738, *p* = 0.009), and the SPEECH (mean difference = 0.818, 95% CI = 0.109/1.608, *p* = 0.039) groups. Paired sample t-tests between T0 and T1 for 1000 bootstrap resamples showed that only the AVG training led to improvements in PD (t-test_(42)_ = −6.622, mean difference = −1.671, 95% CI = −2.152/−1.189, *p* = 0.001); while, the WAIT (t-test_(10)_ = 0.001, mean difference = 0.0001, 95% CI = −0.706/0.706, *p* = 0.971), SNAVG (t-test_(13)_ = −1.394, mean difference = −0.832, 95% CI = −1.895/0.369, *p* = 0.186) and SPEECH (t-test_(10)_ = −1.423, mean difference = −0.588, 95% CI = −1.353/0.176, *p* = 0.205) groups did not. Accordingly, we put the SNAVG and SPEECH groups together in order to create a larger combined active control group (*n* = 22) to test whether the significant effect of AVG upon PD was confirmed. A significantly different individual gain was again found (F_(1,62)_ = 7.987, *p* = 0.006, η_p_^2^ = 0.114; combined control group mean = 0.631, 95% CI = 0.096/1.162), and paired sample t-test for 1000 bootstrap resamples showed no improvements in PD in the combined control group (t-test_(21)_ = −1.033, mean difference = −0.294, 95% CI = −0.824/0.235, *p* = 0.308). Surprisingly, the gain in PD after AVGs training turns out to be more than double compared to that found in the combined active control group (Fig. 2). Moreover, in order to achieve a more similar sample size between the groups, we put the WAIT, SNAVG and SPEECH groups together (*n* = 36) to test whether the significant effect of AVG upon PD was confirmed. A significantly different individual gain was found (F_(1,76)_ = 14.071, *p* < 0.001, η_p_^2^ = 0.156; combined control group mean=0.516, 95% CI = 0.067/0.964), and paired sample t-test for 1000 bootstrap resamples showed no improvements in PD in the combined control group (t-test_(35)_ = −1.745, mean difference = −0.503, 95% CI = −1.060/0.107, *p* = 0.097). These findings support what we found by running the analyses with the WAIT, SNAVG and SPEECH groups separately.

**Fig. 3:**
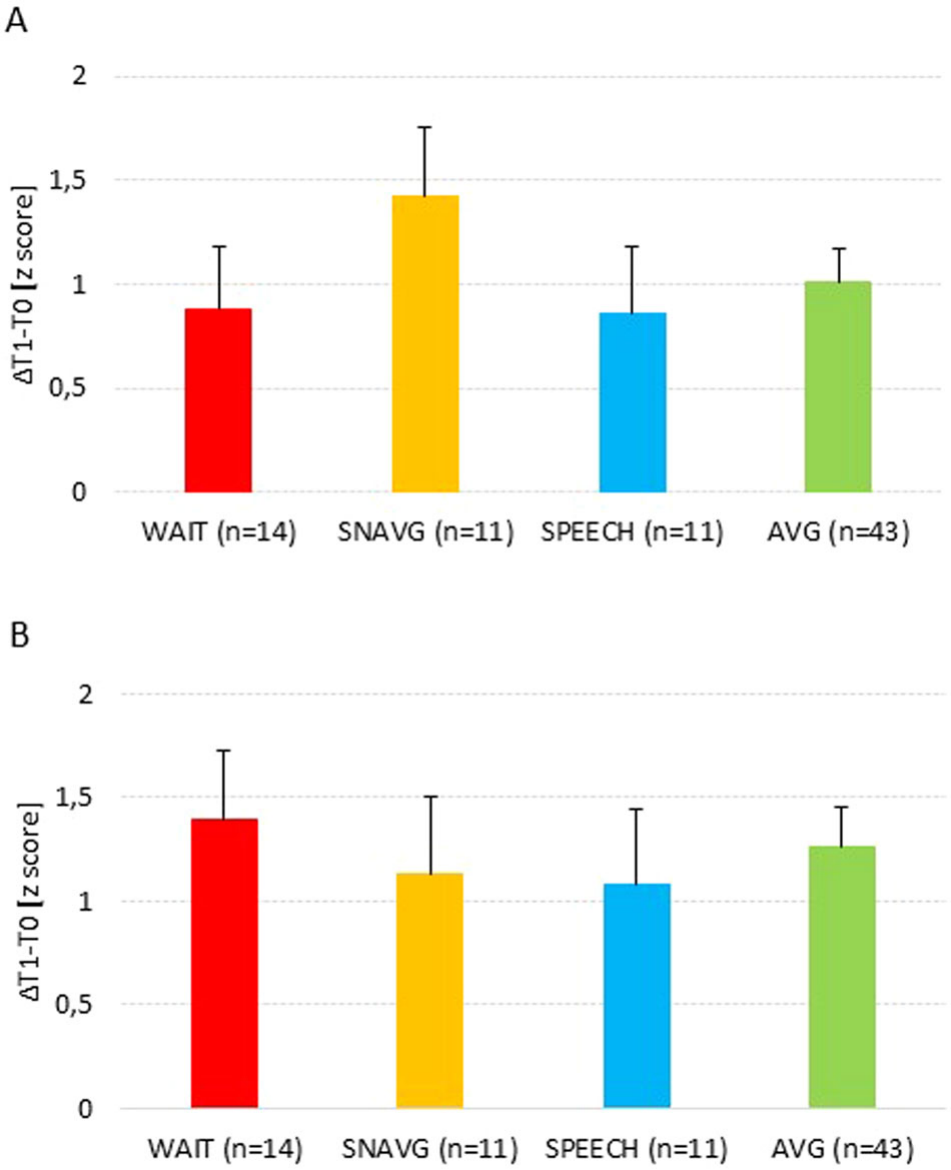
Gains in phonological working memory and RAN. Effects of AVGs upon phonological working memory (**A**) and RAN (**B**). Mean of the individual gain between pre- and post-training (ΔT1-T0). WAIT Waiting list, SNAVG Serious Non-Action Video Game, SPEECH Speech therapy (treatment-as-usual), AVG Action Video game. Error bars represent standard error of the mean.

On the contrary, the training groups did not show any statistically significant difference in individual gain in PWR (F_(3,72)_ = 0.647, *p* = 0.587, η_p_^2^ = 0.026) and in RAN (F_(3,74)_ = 0.172, *p* = 0.915, η_p_^2^ = 0.007) (Fig. 3).

In order to consider individual trials, we implemented alternative statistical approaches such as linear mixed effects models. We therefore ran repeated-measures analyses with time (T0 and T1), group (AVG, SNAVG, SPEECH and WAIT) and time × group as fixed factors, and subject ID as random effects, by using linear mixed effects models. A statistically significant time × group effect was found upon phoneme discrimination (F_(3,75)_ = 3.600, *p* = 0.017), but not upon PWR (F_(3,73.72)_ = 1.032, *p* = 0.384) and RAN (F_(3,75)_ = 0.175, *p* = 0.913). Planned comparisons between T0 and T1 showed that only the AVG training led to improvements in phoneme discrimination (mean difference = 1.671, *p* < 0.001), while, the WAIT (mean difference = 0.832, *p* = 0.070), SNAVG (mean difference = 0.002, *p* = 0.997) and SPEECH (mean difference = 0.590, *p* = 0.252) groups did not.

### Catch-up in phonemic awareness within the AVG group

The planned comparison between AVG and not-at-risk children showed that there was no difference in PD scores at T1 for 1000 bootstrap resamples (t-test_(82)_ = 1.608, mean difference = 0.417, 95% CI = −0.077/0.908, *p* = 0.126). This result suggests that AVG improves phonemic awareness making it comparable to those of not-at-risk children.

### Analysis of phonemic awareness individual data within the AVG group

We demonstrated that AVG selectively improves phonemic awareness in pre-readers at risk for DD. However, one important question needs to be addressed: How frequent is this improvement among at-risk pre-readers after AVG? We therefore compared the individual gain in PD between the AVG group and the combined control group. More than 80% of at-risk pre-readers within the AVG group showed a gain in PD which was above the mean gain observed in the combined control group. This result suggests that this unconventional training (i.e., AVG) has a high frequency of efficacy upon deficits in the phonemic awareness.

### The long-lasting effect of AVGs upon phonemic awareness

**Fig. 4:**
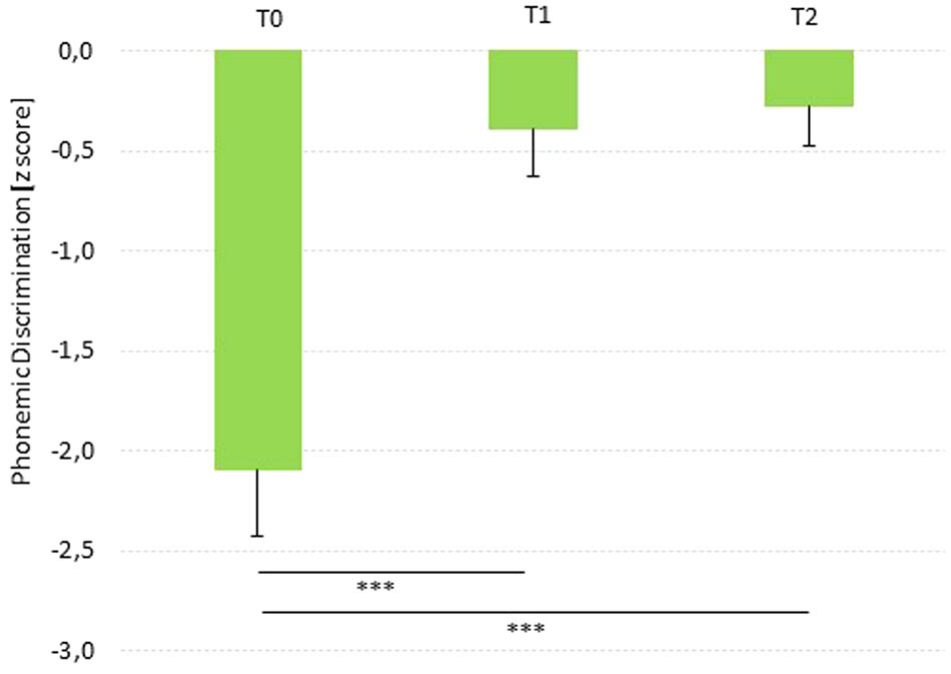
The long-lasting effect of AVGs upon phonemic awareness. Mean in PD at the different time points (*n* = 33), i.e., pre-AVG (T0), post-AVG (T1) and 6-months follow-up (T2). Error bars represent standard error of the mean. ***p < 0.01.

Finally, 33 children within the AVG group took part in the follow-up session. The paired sample t-test upon PD for 1000 bootstrap resamples revealed that there are no statistically significant differences between the post-training (mean = −0.390, SD = 1.356) and 6-months follow-up (mean = −0.272, SD = 1.179) sessions (*t*-test_(32)_ = −0.494, mean difference = −0.118, 95% CI = −0.626/0.294, *p* = 0.626). That is, the improvement in the phonemic awareness induced through AVG was maintained after 6 months following the end of the AVG training. Moreover, paired sample t-tests between T0 and T1 and T0 and T2 for 1000 bootstrap resamples showed significant improvements in PD also in this sub-sample (T0-T1: t-test_(32)_ = −5.889, mean difference = −1.706, 95% CI = −2.265/−1.109, *p* = 0.001; and, T0-T2: t-test_(32)_ = −6.336, mean difference = −1.824, 95% CI = −2.372/−1.221, *p* = 0.001) (Fig. 4).

## Discussion

The causal link between attentional control and reading development has already been provided by studies using AVG, traditional and game-based executive functions training programs in (a)typical readers^6,17,36,61–63,69^. To the best of our knowledge, however, no previous study has used AVG training to improve the reading-related predictors in pre-readers at risk of DD. This prevention study reports the effects of a commercial AVG upon consolidated reading development predictors in pre-readers at-risk for DD. Children at-risk and not-at-risk for DD were selected on the basis of their skills in three reading-related core tasks, i.e. phonemic awareness, phonological working memory and RAN. To investigate the possible role of an AVG training on the most relevant causal factors of DD, we compared its effect to both a passive control group (WAIT) and two active control groups (i.e., SNAVG and SPEECH), following the gold standard methodology for behavioural interventions^91^. The used AVG includes several mini-games loading high-speed events and fast-moving targets on the peripheral visual field, and spatial and temporal unpredictability with high perceptual and motor loads which are hypothesised to enhance the multisensory evidence accumulation^20^.

Overall, our results demonstrate far-transfer, but specific phonological benefits following this unconventional training as compared to serious games (i.e., SNAVG) and the treatment-as-usual for DD (i.e., SPEECH). Indeed, the effects of the AVG training are not general-domain on phonological working memory and rapid naming, pointing to the neurocognitive circuitry underlying phonemic awareness as a specific target for AVG in at-risk pre-readers. None of the AVG training activities loaded auditory-phonological processing as in the phonemic awareness. Children in the AVG group showed greater enhancements in phonemic awareness as compared to the control-untrained (WAIT) and control-trained groups (SPEECH and SNAVG), but not in phonological working memory and rapid naming. Perhaps more important, this far-transfer plastic change led to a full recovery in phonemic awareness when compared to the not-at-risk pre-readers. Moreover, this enhancement was maintained 6 months after the end of AVG training. Finally, phonemic awareness benefits were observed in more than 80% of pre-readers in the AVG group, thus showing further clinical relevance of this child-friendly and unconventional remediation program. On the contrary, neither SPEECH nor SNAVG nor WAIT showed statistically significant enhancements in phonemic awareness.

By showing significant effects upon phonemic awareness in pre-readers at-risk for DD, our results extend previous data showing AVG-induced effects upon auditory-phonological skills in both healthy adults^20,23^ and in children with DD^6,35,69,70^. Similarly, Nava et al.^92^ have shown that only 2-weeks training with commercial action-like mini-games can promote optimal multisensory integration and visuo-spatial enhancements in children aged 4-5 years, inducing long-term plastic changes in the developing brain at least up to 3 months.

It is therefore plausible to hypothesise that the AVGs accelerate the multisensory “accumulation of evidence”^20^ leading to a reduction of the sluggish attentional shifting^77^ during the pre-reading phase too. The neural circuits involved in the “accumulation of evidence” might be shared across modalities^20^. An efficient functioning in the auditory “accumulation of evidence” and the rapid attentional shifting^28,29,77,83,84,86^ is crucial for the development of adequate phonemic discrimination skills^41,44,46,51^ which are causally linked to reading acquisition^38–40,42^. Phonemic awareness skills in Italian children attending kindergarten have been shown to be a reliable marker to identify at-risk pre-readers and to predict future reading skills during the first grades of primary school^32,35,36^. Rapid auditory processing^46^ and timely attentional shifting^45^ are both required to develop efficient phonemic awareness skills. The neural correlates of these sensory and attentional functions can be linked to the right-lateralized neural networks underlying the alerting system^74^, the ventral stimulus-driven control of attention^10^, the “When” system^73^, temporal-sampling processing^41^ or salience acoustic processing^75^. Interestingly, these right-lateralized neural networks can overlap with those underpinning the multisensory accumulation of evidence. Nevertheless, it is important to note that the auditory cortices involved in phonemic awareness receives information not only from these multisensory networks, but also from lateral projections from primary and secondary visual cortices and from feedforward inputs from nonspecific and higher order thalamic regions (e.g., suprageniculate, posterior, anterior dorsal and magnocellular divisions of the medial geniculate complex, and portions of the pulvinar complex)^93,94^.

The far-transfer, specific benefits in phonemic awareness following AVGs do not agree with previous findings showing significant effects of the AVG training upon phonological working memory^35,69,70^ and RAN^71^. However, other studies provided similar results in children with DD^6,72^ and adult typical readers^15,16^. The null effects of AVGs upon phonological working memory and RAN, could be due to the fact that the neural networks underlying these more complex functions are still developing in children aged 5-years^95,96^. Moreover, these null results suggest that AVGs may specifically affect the right-lateralized feed-forward and stimulus-driven neural networks underlying phonemic awareness in pre-readers, but not the bilateral top-down fronto-parietal^97^ and the left reading ventral and dorsal networks^56,98^ which are known to be involved in working memory and visual access to phonological representations, respectively. Taken together, these results agree with recent data supporting generalised benefits in untrained tasks and everyday cognitive functioning provided by programs training speed of processing, such as “accumulation of evidence”^99–101^.

Finally, our results showed that other active training (i.e., SPEECH and SNAVG) did not lead to significant enhancements in phonological core tasks. Although speech therapy represents the training-as-usual for at-risk pre-readers, it does not seem efficiently to affect some of the mechanisms (i.e., reward mechanisms and/or attentional control) which are crucial for a rapid processing of information^10,11^ and for learning^9^. Moreover, it is well-known that the effects of speech therapy can be detected only after a longer period of time^62^. Similarly, the SNAVG are not able to adequately stimulate the attentional control^9^. As stimulating stimulus-driven and/or goal-directed control of attention accelerates the attentional shifting^29,45,86^ and enhances the speed of accumulation of sensory evidence^20,83,84^, this can explain the lack of efficacy of the SNAVG.

Notwithstanding the novelty of the present results, they need to be considered within the limits of the study. First, the sample size of the SPEECH, SNAVG and WAIT groups is limited. Nevertheless, our results are confirmed also when the AVG group was compared to the larger combined active control group. However, further studies in larger samples are encouraged. Second, the criterion adopted for identifying pre-readers at-risk for DD (i.e., at least −1.00 SD below the mean in at least one of the selected reading-related tasks) could appear quite liberal. However, the inclusion of participants with mild impairments prevented us from the regression to the mean effect. Indeed, if participants of an intervention program are selected because they are particularly low on a specific criterion variable, scores on the same or related variables tend to regress toward the mean at a later measurement occasion^102^. Third, we did a follow-up assessment after 6 months at the end of the training. This did not allow us to monitor the development of reading skills during the early years of primary school. However, previous longitudinal prospective studies showed that pre-readers with efficient phonemic awareness will develop good reading skills during the first years of primary school^32,34–36,40^. Fourth, we did not collect data about the neural correlates of the AVGs’ effects on phonemic awareness. As our neuropsychological results suggested that AVGs may specifically affect the right-lateralized feed-forward and stimulus-driven neural networks of attentional control, future studies are needed to test this neurobiological hypothesis. Fifth, as the intensity of the SPEECH program differed compared to those of the AVG and SNAVG groups, a greater effect of spontaneous development may be possible in the former group. Future studies are needed in order to assess the potential effects of different remediation programs’ intensities upon reading-related skills development. Sixth, further prevention studies with longer-term follow-ups and larger control group sample sizes, are required to provide a more comprehensive understanding of the extent of future reading development.

The existence of specific, temporal windows of opportunity during which environmental factors can prevent the risk for impairment is increasingly acknowledged but seldom addressed empirically^103^. Our results showed that AVGs induce a large, long-lasting and clinically relevant effect on the tuning of phonological representations in pre-readers at-risk for DD, by accelerating the sampling rate of sensory evidence and reducing the sluggish attentional shifting. According to these findings, AVG could be leveraged for preventing multisensory processing difficulties in several neurodevelopmental disorders characterised by an early dysfunction of attentional deployment, such as DD^37,55^, attention deficit and hyperactivity disorder^25^, developmental coordination disorder^5^, and autism spectrum disorder^104^, as well as in typically developing children^17,92^. Implicit in these discoveries is the future potential for targeted manipulation of critical period timing to optimise the impact of preventive interventions^105^.

## Methods

### Participants

One hundred twenty pre-readers attending the last year of kindergarten (57 females and 63 males, mean age=5.58 years, SD = 0.43) were recruited at the Scientific Institute, IRCCS “Eugenio Medea” (Bosisio Parini, Italy). All participants had a typical intelligence quotient (as estimated by the Block design subtest of the WPPSI, mean=12.08, SD = 3.47) and no neurological, sensorial or psychiatric diseases.

Among the recruited participants, children were considered at-risk for DD whether they obtained a score below −1.00 SD in at least one of the following reading-related tasks: phonemic awareness, phonological working memory or RAN. According to this criteria, 79 pre-readers were classified as at-risk for DD (see Table 2). All the children’s parents gave written informed consent after a description of the research study, in accordance with the principles of the Declaration of Helsinki; the ethics committee of the Scientific Institute “Eugenio Medea” approved the research protocol.

**Table 2:** Classification of the at-risk children

Performance	Phonological task	*N*
One test below −1.00 SD	PD	10
	PWR	48
	RAN	5
Two tests below −1.00 SD	PD and PWR	10
	PD and RAN	1
	PWR and RAN	3
Three tests below −1.00 SD	PD, PWR and RAN	2

*PD* Phonemic discrimination, *PWR* Pseudo-word repetition, *RAN* Serial rapid automatized naming.

### Study design

The AVG, SNAVG and SPEECH groups underwent an assessment administered by a neuropsychologist before (T0) and at the end of the training (T1) in a dimly lit and a quiet room. Regarding the not-at-risk children and the WAIT group, they underwent the neuropsychological assessment two times after a comparable time interval of AVG, SNAVG and SPEECH (i.e., 1.5–2.5 months) to control for spontaneous development and possible test-retest effects^91^. Moreover, the AVG group was also tested after 6 months from the end of the training (T2).

To control for possible experimenter effects, the experimenters carrying out the different training programs were different from those who assessed children before and after them. In addition, experimenters assessing children before and after the training programs, did not know in which training group the child was included.

A priori power calculations were conducted using GPower^106^ to estimate the smallest sample size needed to detect a medium effect size^27^ with 80% statistical power. The analysis was modelled for a repeated measure ANOVA, four groups with two measurements, alpha equal to 0.05. Under these assumptions, the minimal sample size predicted to be needed with 80% statistical power was 48 subjects.

### Procedures

#### Reading-related neuropsychological assessment

1. Phonemic awareness^107^
  Phonemic awareness was measured by using the PD task. Stimuli were composed by 15 pairs of bisyllabic pseudowords differing only by one phoneme determining a phonemic contrast between the two sounds (e.g., “pado” and “fado”, “leta” and “leda”). In order to avoid mouth reading, the experimenter read each pair of pseudowords covering the mouth. Children were asked to judge whether the pseudowords within each pair were identical or different. Accuracy (i.e., number of correct answers) was recorded and used as the dependent variable.
2. Phonological working memory^108^
  Phonological working memory was measured by using a PWR task. Stimuli were composed by 40 pseudowords of differing syllable length (10 bisyllabic, 10 tri-syllabics, 10 forth-syllabics and 10 polysyllabic) and complexity of sound combinations. Children were asked to repeat each item as accurately as possible. If the child did not hear or understand the pseudoword, he/she was encouraged to repeat what he/she listened. Accuracy (i.e., number of correct answers) was collected and used as the dependent variable.
3. RAN^32^
  Visual to spoken mapping was measured by using a serial RAN task. We used RAN of colours to control possible confounding effects associated with exposure to alphanumeric stimuli. Stimuli consisted of a sequence of eight filled coloured circles (i.e., red, blue, green and yellow). The participants were asked to name the colours as fast as possible. Both speed (seconds) and accuracy (number of errors) were recorded. Since accuracy showed a ceiling effect, the dependent variable was the speed (in seconds).

### Intervention treatments

At-risk children were assigned to different groups, i.e., AVG, WAIT, SNAVG, and SPEECH using an inequality randomization. All participants were not aware of the training programs used by the other groups.

#### AVG

The commercial game “Space Invaders Extreme 2” was used as it has all mechanisms characterising AVG (i.e., presentation of multiple peripheral, rapidly moving, spatiotemporally unpredictable stimuli^8,9^). The game was played on a Nintendo DS^®^ console characterised by two screens from which the player can monitor the movement of the enemies and of his/her spaceship. The aim of the game was to fight against enemies and to avoid them reaching the planet. Its auditory environment was characterised by electronic background sounds and by specific electronic sounds (e.g., explosions) every time the spaceshift hit enemies or was hit by enemies. This game required accurate spatial and temporal attention to pass to the subsequent level. The game increased the difficulty by adapting to the player’s skills. Children were divided into small sub-groups composed of three children and supervised by a neuropsychologist. Each child played at least 20 sessions of 45 min (mean = 26, SD = 4.93), four times per week, distributed over 1.5 months.

#### SNAVG

The SNAVG group was asked to play at different serious minigames as previously described in Gaggi et al.^109^. Each minigame is designed to train specific skills linked to grapheme-to-phoneme mapping, rapid auditory discrimination and visuo-spatial attention, without any mechanisms characterising AVG. The minigames increased the difficulty by adapting to the player’s skills. Children were divided into small sub-groups composed of three children and supervised by a neuropsychologist. Each child played at least 25 sessions of 45 min (mean = 26.5, SD = 0.40), four times per week, distributed over 1.5 months.

#### SPEECH

The SPEECH group underwent phonological training with a speech therapist, which represents the training-as-usual for pre-readers at-risk for DD. The focus of the SPEECH therapy is to strengthen skills in phonological awareness (i.e., phoneme deletion, phoneme counting, phoneme blending, syllable segmentation, rhyme oddity, and rhyme judgement) and in automatizing grapheme-to-phoneme mapping. Each child attended at least 21 individual sessions of 45 min (mean = 29.47, SD = 8.67), distributed over 3.7 months. The frequency of the training-as-usual is clinically fixed and scheduled for two times per week. A greater effect of spontaneous development may be possible in this group.

### Statistical analysis

Z-scores for each reading-related task were calculated according to the mean and SD observed in the not-at-risk group. The individual gain between pre- and post-training (ΔT1-T0) in each reading-related task for each participant was calculated as the difference between z-scores. To evaluate the effects of and to compare performance among the different training programs (i.e., AVG, WAIT, SNAVG and SPEECH), we ran a univariate analysis of the covariance (ANCOVA) on each reading-related skill with training group as between variable, ΔT1-T0 as dependent variable, and the individual’s score at T0 as covariate. The percentage of pre-readers in the AVG group who showed an amelioration above those expected by observing the combined at-risk group (i.e., WAIT, SNAVG and SPEECH), was estimated in order to assess its clinical relevance. Finally, the AVG group was tested after 6 months from the end of the training to evaluate long-lasting effects.

### Reporting summary

Further information on research design is available in the Nature Research Reporting Summary linked to this article.

### Supplementary information

Reporting summary

## Data Availability

Data are available upon requests from the corresponding authors.
