# Supplementary material for: Action video games normalise the phonemic awareness in pre-readers at risk for developmental dyslexia
Source: NPJ Sci Learn. 2024 Mar 21;9:25. doi: 10.1038/s41539-024-00230-0 (PMC10957868; doi:10.1038/s41539-024-00230-0)
Supplement: Supplementary file 1 — Reporting summary [file 41539_2024_230_MOESM1_ESM.pdf]

Corresponding author(s): Andrea Facoetti
Sara Bertoni

Last updated by author(s): Feb 20, 2024

Reporting Summary

Nature Portfolio wishes to improve the reproducibility of the work that we publish. This form provides structure for consistency and transparency in reporting. For further information on Nature Portfolio policies, see our Editorial Policies and the Editorial Policy Checklist.

Statistics

For all statistical analyses, confirm that the following items are present in the figure legend, table legend, main text, or Methods section.

n/a	Confirmed	
☐	☒	The exact sample size (*n*) for each experimental group/condition, given as a discrete number and unit of measurement
☐	☒	A statement on whether measurements were taken from distinct samples or whether the same sample was measured repeatedly
☐	☒	The statistical test(s) used AND whether they are one- or two-sided<br>*Only common tests should be described solely by name; describe more complex techniques in the Methods section.*
☐	☒	A description of all covariates tested
☐	☒	A description of any assumptions or corrections, such as tests of normality and adjustment for multiple comparisons
☐	☒	A full description of the statistical parameters including central tendency (e.g. means) or other basic estimates (e.g. regression coefficient) AND variation (e.g. standard deviation) or associated estimates of uncertainty (e.g. confidence intervals)
☐	☒	For null hypothesis testing, the test statistic (e.g. *F*, *t*, *r*) with confidence intervals, effect sizes, degrees of freedom and *P* value noted<br>*Give P values as exact values whenever suitable.*
☒	☐	For Bayesian analysis, information on the choice of priors and Markov chain Monte Carlo settings
☒	☐	For hierarchical and complex designs, identification of the appropriate level for tests and full reporting of outcomes
☐	☒	Estimates of effect sizes (e.g. Cohen's *d*, Pearson's *r*), indicating how they were calculated

Our web collection on statistics for biologists contains articles on many of the points above.

Software and code

Policy information about availability of computer code

Data collection	Excel - Microsoft Office 15
Data analysis	IBM SPSS Statistics 26

For manuscripts utilizing custom algorithms or software that are central to the research but not yet described in published literature, software must be made available to editors and reviewers. We strongly encourage code deposition in a community repository (e.g. GitHub). See the Nature Portfolio guidelines for submitting code & software for further information.

Data

Policy information about availability of data

All manuscripts must include a data availability statement. This statement should provide the following information, where applicable:

- Accession codes, unique identifiers, or web links for publicly available datasets
- A description of any restrictions on data availability
- For clinical datasets or third party data, please ensure that the statement adheres to our policy

Data (and code) are only available upon request to the corresponding author.

Research involving human participants, their data, or biological material

Policy information about studies with human participants or human data. See also policy information about sex, gender (identity/presentation), and sexual orientation and race, ethnicity and racism.

Reporting on sex and gender	Sex was assigned. We verified that our samples did not differ for 'sex'
Reporting on race, ethnicity, or other socially relevant groupings	n/a
Population characteristics	We provide information about age, IQ, risk for language and learning-disorders, and experimental training programs
Recruitment	Participants attending the last year of kindergarten were recruited at the Scientific Institute, IRCCS "Eugenio Medea" (Bosisio Parini, Italy).
Ethics oversight	The ethics committee of the Scientific Institute Eugenio Medea approved the research protocol

Note that full information on the approval of the study protocol must also be provided in the manuscript.

Field-specific reporting

Please select the one below that is the best fit for your research. If you are not sure, read the appropriate sections before making your selection.

☐ Life sciences ☒ Behavioural & social sciences ☐ Ecological, evolutionary & environmental sciences

For a reference copy of the document with all sections, see nature.com/documents/nr-reporting-summary-flat.pdf

Behavioural & social sciences study design

All studies must disclose on these points even when the disclosure is negative.

Study description

The AVG, SNAVG and SPEECH groups underwent an assessment administered by a neuropsychologist before (T0) and at the end of the training (T1) in a dimly lit and a quiet room. Regarding the not-at-risk children and the WAIT group, they underwent the neuropsychological assessment two times after a comparable time interval of AVG, SNAVG and SPEECH (i.e., 1.5-2.5 months) to control for spontaneous development and possible test-retest effects (Green et al., 2019). Moreover, the AVG group was also tested after six months from the end of the training (T2).
To control for possible experimenter effects, the experimenters carrying out the different training programs were different from those who assessed children before and after them. In addition, experimenters assessing children before and after the training programs, did not know in which training group the child was included.

Research sample

Among the recruited participants (N=120), children were considered at-risk for DD whether they obtained a score below -1.00 SD in at least one of the following reading-related tasks: phonemic awareness, phonological working memory or RAN. According to this criteria, 79 pre-readers were classified as at-risk for DD. At-risk children were assigned to different groups, i.e. AVG, WAIT, SNAVG, and SPEECH using an inequality randomization. All participants were not aware of the training programs used by the other groups.

Sampling strategy

A priori power calculations were conducted using GPower (Erdfelder et al., 1996) to estimate the smallest sample size needed to detect a medium effect size (Puccio et al., 2023) with 80% statistical power. The analysis was modelled for a repeated measure ANOVA, four groups with two measurements, alpha equal to 0.05. Under these assumptions, the minimal sample size predicted to be needed with 80% statistical power was 48 subjects.

Data collection

Reading-related neuropsychological assessment
1. Phonemic awareness (Marotta et al., 2004)
Phonemic awareness was measured by using the PD task. Stimuli were composed by 15 pairs of bisyllabic pseudowords differing only by one phoneme determining a phonemic contrast between the two sounds (e.g., "pado" and "fado", "leta" and "leda"). In order to avoid mouth reading, the experimenter read each pair of pseudowords covering the mouth. Children were asked to judge whether the pseudowords within each pair were identical or different. Accuracy (i.e., number of correct answers) was recorded and used as the dependent variable.
2. Phonological working memory (Bertelli & Bilancia, 2006)
Phonological working memory was measured by using a PWR task. Stimuli were composed by 40 pseudowords of differing syllable length (10 bisyllabic, 10 tri-syllabics, 10 forth-syllabics and 10 polysyllabic) and complexity of sound combinations. Children were asked to repeat each item as accurately as possible. In order to avoid mouth reading, the experimenter read each pair of pseudowords covering the mouth. If the child did not hear or understand the pseudoword, he/she was encouraged to repeat what he/she listened. Accuracy (i.e., number of correct answers) was collected and used as the dependent variable.
3. RAN (Franceschini et al., 2012)
Visual to spoken mapping was measured by using a serial RAN task. We used RAN of colours to control possible confounding effects associated with exposure to alphanumeric stimuli. Stimuli consisted of a sequence of eight filled coloured circles (i.e., red, blue, green and yellow). The participants were asked to name the colours as fast as possible. Both speed (seconds) and accuracy (number of errors) were recorded. Since accuracy showed a ceiling effect, the dependent variable was the speed (in seconds).

At-risk children were assigned to different groups, i.e. AVG, WAIT, SNAVG, and SPEECH using an inequality randomization. All participants were not aware of the training programs used by the other groups.
1)) AVG
The commercial game "Space Invaders Extreme 2" was used as it has all mechanisms characterising AVG (i.e., presentation of multiple peripheral, rapidly moving, spatiotemporally unpredictable stimuli; Green & Bavelier, 2012; Bavelier & Green, 2019). The game was played on a Nintendo DS® console characterised by two screens from which the player can monitor the movement of the enemies and of his/her spaceship. The aim of the game was to fight against enemies and to avoid them reaching the planet. Its auditory environment was characterised by electronic background sounds and by specific electronic sounds (e.g., explosions) every time the spaceshift hit enemies or was hit by enemies. This game required accurate spatial and temporal attention to pass to the subsequent level. The game increased the difficulty by adapting to the player's skills. Children were divided into small sub-groups composed of three children and supervised by a neuropsychologist. Each child played at least 20 sessions of 45 minutes (mean=26, SD=4.93), four times per week, distributed over 1.5 months.
2) SNAVG
The SNAVG group was asked to play at different serious minigames as previously described (Gaggi et al., 2017). Each minigame is designed to train specific skills linked to grapheme-to-phoneme mapping, rapid auditory discrimination and visuo-spatial attention, without any mechanisms characterising AVG. The minigames increased the difficulty by adapting to the player's skills. Children were divided into small sub-groups composed of three children and supervised by a neuropsychologist. Each child played at least 25 sessions of 45 minutes (mean=26.5, SD=0.40), four times per week, distributed over 1.5 months.
3) SPEECH
The SPEECH group underwent phonological training with a speech therapist, which represents the training-as-usual for pre-readers at-risk for DD. The focus of the SPEECH therapy is to strengthen skills in phonological awareness (i.e., phoneme deletion, phoneme counting, phoneme blending, syllable segmentation, rhyme oddity, and rhyme judgement) and in automatizing grapheme-to-phoneme mapping. Each child attended at least 21 individual sessions of 45 minutes (mean=29.47, SD=8.67), distributed over 3.7 months. The frequency of the training-as-usual is clinically fixed and scheduled for two sessions per week. A greater effect of spontaneous development may be possible in this group.

To control for possible experimenter effects, the experimenters carrying out the different training programs were different from those who assessed children before and after them. In addition, experimenters assessing children before and after the training programs, did not know in which training group the child was included.

Timing	START: March 2018<br>END: November 2018
Data exclusions	No data were excluded from the analyses
Non-participation	Ten participants dropped because of familial logistic issues
Randomization	Inequality randomization

Reporting for specific materials, systems and methods

We require information from authors about some types of materials, experimental systems and methods used in many studies. Here, indicate whether each material, system or method listed is relevant to your study. If you are not sure if a list item applies to your research, read the appropriate section before selecting a response.

Materials & experimental systems

n/a	Involved in the study
☒	☐ Antibodies
☒	☐ Eukaryotic cell lines
☒	☐ Palaeontology and archaeology
☒	☐ Animals and other organisms
☒	☐ Clinical data
☒	☐ Dual use research of concern
☒	☐ Plants

Methods

n/a	Involved in the study
☒	☐ ChIP-seq
☒	☐ Flow cytometry
☒	☐ MRI-based neuroimaging

Plants

Seed stocks

Report on the source of all seed stocks or other plant material used. If applicable, state the seed stock centre and catalogue number. If plant specimens were collected from the field, describe the collection location, date and sampling procedures.

Novel plant genotypes

Describe the methods by which all novel plant genotypes were produced. This includes those generated by transgenic approaches, gene editing, chemical/radiation-based mutagenesis and hybridization. For transgenic lines, describe the transformation method, the number of independent lines analyzed and the generation upon which experiments were performed. For gene-edited lines, describe the editor used, the endogenous sequence targeted for editing, the targeting guide RNA sequence (if applicable) and how the editor was applied.

Authentication

Describe any authentication procedures for each seed stock used or novel genotype generated. Describe any experiments used to assess the effect of a mutation and, where applicable, how potential secondary effects (e.g. second site T-DNA insertions, mosiacism, off-target gene editing) were examined.
